# The power of concentration: Antipredator responses to diluted frozen crayfish alarm cues provide insights on ecologically relevant concentrations and updates to methodology

**DOI:** 10.1371/journal.pone.0340001

**Published:** 2025-12-31

**Authors:** Gabrielle H. Achtymichuk, Kaitlyn M. Fish, Maud C. O. Ferrari

**Affiliations:** 1 Department of Veterinary Biomedical Sciences, WCVM, University of Saskatchewan, Saskatoon, Saskatchewan; 2 Department of Biology, University of Saskatchewan, Saskatoon, Saskatchewan; IEAPM: Instituto de Estudos do Mar Almirante Paulo Moreira, BRAZIL

## Abstract

To optimize fitness when facing predation, animals perform threat-sensitive predator avoidance whereby they match the magnitude of the antipredator response to the severity of the perceived threat. Injury-released chemical alarm cues are a reliable indicator of predation risk in aquatic organisms, triggering overt antipredator responses upon detection by conspecifics. Animals with threat sensitivity typically have graded responses to increasing concentrations of these cues which plateau when a maximal response is reached, however, this is undocumented in crayfish. Furthermore, most research currently uses alarm cue exposures consisting of one crushed crayfish diluted in 100–400 mL of water, while it could be ecologically relevant for them to respond to lower concentrations, especially given that predation events can consist of bites or lacerations which would release less alarm cues. The quantity of cue administered into the tank (exposure concentration) is also highly variable, making experimental comparisons difficult. In our study, we collected crayfish alarm cues by rinsing five cut sites (to mimic laceration wounds) and diluting the cues in 100 mL to 100 L of water. Over two experiments, we determined the antipredator response of crayfish exposed to one of five alarm cue concentrations or a water control. During these trials, 20 mL of the cues were administered into 10 L of water, thereby standardizing the test subject’s exposure to the cues. While we failed to find evidence of graded responses, we discovered that alarm cues elicited overt antipredator behaviour when diluted in up to 10 L of water, but this response was lost when cues were diluted in 100 L. Furthermore, this study is the first to successfully use frozen-thawed alarm cues in crayfish. These findings can help direct future research, providing information on the ecologically relevant range of chemical cues and improving welfare by reducing lab-animal sacrifice.

## Introduction

One mechanism that aquatic organisms use to identify and respond to threats is through the detection of alarm cues – chemical compounds that are released following injury to an individual [for a review, see 1]. Since the release of alarm cues is dependent on mechanical damage to an organism, these cues are a reliable indicator of risk. When detected by nearby conspecifics, alarm cues initiate an immediate antipredator response as well as a wide range of behavioural, morphological, and life-history adaptations, all of which increase a prey’s chances of surviving predators. Common alarm cue induced behavioural responses across aquatic taxa include freezing, increased shelter use, and area avoidance [2–7]. Additionally, shoaling and bottom-dwelling behaviour has been observed in fishes [2,8] while defensive chelae displays can occur in crayfish [3]. Some species also alter their morphology in response to alarm cues, as is the case with some cladoceran crustaceans (e.g., daphnia) which develop thick helmet-like structures and tail spines for antipredator defense [9]. Similarly, alarm cues cause tadpoles to develop a deeper tail and shorter body as this shape improves predator escape [10]. Finally, some species (e.g., fishes, tadpoles) can also alter the timing of major life history events, including hatching and metamorphosis, in response to alarm cues in order to reduce predation risk [11–13].

In addition to triggering overt antipredator responses, alarm cues are essential for long-term learning of potential threats. When cues from an unknown novel predator (i.e., sight, sound, or odour) are detected for the first time (conditioned stimulus) in the presence of alarm cues (innate trigger of fear, unconditioned stimulus), the naïve prey will associate the novel predator as the source of risk through a Pavlovian-like fear conditioning [14]. In subsequent encounters, that prey will recognize the predator as risky and an antipredator response will be triggered upon detection of the predator cue alone – a beneficial response for survival as early detection can increase predator avoidance [14].

In alignment with the threat sensitive predator avoidance hypothesis, these alarm cue responses should follow a gradient, with increasing concentrations representing an increased risk, thus eliciting increased antipredator responses [1,15]. Specifically, a high alarm cue concentration likely represents a predation event that is closer temporally or spatially, therefore the individual is at greater risk of encountering the predator and a large antipredator response is advantageous [1]. However, all antipredator responses incur a cost, whether it is energy allocated into morphological defenses leaving less for growth or reproduction or decreased time spent foraging as individuals freeze or hide [10,15,16]. Therefore, the graded response of the threat sensitive predator avoidance theory is adaptive as it allows individuals to optimize their fitness as they match the expression of their antipredator responses to the magnitude of the perceived risk, thereby avoiding unnecessary costs [15]. This adaptive significance is supported as it is persists across various aquatic taxa, including fishes [17,18], amphibians [10,16,19,20], and larval insects [21]. However, there are exceptions as some species of fishes are reported to have ungraded all-or-nothing responses to alarm cues [22–24]. These animals exhibit a maximal response to the cues once a certain concentration threshold is surpassed [22–24].

Responding to high concentrations of alarm cues with a large antipredator response is intuitively advantageous for prey species because there is a greater probability that they will encounter the predator (i.e., temporally or spatially closer). Nonetheless, responding to low concentrations of alarm cues is also beneficial. This is because, in nature, predation events do not only consist of whole-body damage and consumption by the predator (i.e., successful predation) but also include single laceration or bite wounds with the prey subsequently escaping before further damage (i.e., unsuccessful predation) [25,26]. In these latter cases, very little alarm cue would be released, and these cues would be further diluted in the surrounding body of water. It would be beneficial for conspecifics to have evolved to respond to these low concentrations and respond accordingly to avoid predation. Furthermore, these low concentrations are often more interesting to researchers, as it allows examination of subtle trade-offs involving antipredator responses. For example, starvation, increased body size, and an older age, are all factors that can cause individuals to shift their behaviour, minimizing their antipredator responses to alarm cues in favor of foraging [24,27–30]. However, if the risk (i.e., alarm cue concentration) becomes too high, these behavioural changes might not be observed, as the benefits of foraging over fear responses can no longer outweigh the costs.

While the threat sensitive predator hypothesis results in a graded response to predation-related cues such as alarm cues, it is also common for these graded responses to reach a limit and plateau [10,31–33]. One mechanism by which this plateau could occur is if the antipredator response reaches a physical or physiological limit [10,34]. Alternately, antipredator responses could reach a plateau as the cost incurred by expressing the response becomes higher than the benefits of predator avoidance [10,34,35]. Regardless of the mechanism, species that are thought to have an ungraded all-or-nothing threshold response to predation-related cues may simply have not been tested with concentrations on a fine enough scale to detect the gradation in their antipredator responses and instead made observations in the response plateau range [10,36]. This has been found to be true for many species ([33,36–40], but see [22–24] for exceptions).Since it is well-established that the alarm cue concentration can influence the resulting antipredator response, it is an important factor for researchers to consider when designing experiments and concluding on results. It is especially critical to ensure the proper conclusions are drawn when making comparisons across studies, or when extrapolating laboratory experiments to natural populations, as individuals should be exposed to threat levels (i.e., concentrations) that are equivalent to those they would experience in their natural environment. However, this is a particular challenge with alarm cues because, despite being an important aquatic communication system and well studied since the mid 1900s [1,41], there are still many unanswered questions about these cues. This is likely because alarm cues are documented in a wide range of taxa, from corals and crustaceans to fishes and amphibians [1], and the cues are not conserved across species. For example, in Ostariophysi fishes, alarm cues are known to be produced from club cells located in the epidermis, with the cues hypothesized to be released upon mechanical rupture of these cells [42]. In contrast, alarm cues are located in the hemolymph in invertebrates such as crustaceans [43,44] and some gastropods [45, for an exception, see 46], but the exact production mechanism is unknown as club cells have not been identified in these species [44]. Furthermore, alarm cues are primarily species specific – individuals innately display antipredator responses to conspecific alarm cues, and only generalize these responses to closely-related heterospecific alarm cues [47–49]. Responses to distantly-related alarm cues are documented, but those are the results of learned alarm cue recognition between sympatric prey-guild species [50,51].

The aforementioned species-specificity and large variation in the origin of alarm cues across taxa supports the current accepted theory that the chemical structures and properties of alarm cues differ across species [1]. Despite this knowledge, the exact chemical composition of any alarm cues remains currently undocumented. Attempts have been made to identify the active molecules of alarm cues in fishes [52–55], amphibians [56–58], crustaceans and gastropods [44–46], and cnidarians [59], however results remain inconclusive and are often contradictory. Differences in the chemical composition of these cues might however explain the different chemical properties of heterospecific alarm cues, including why alarm cues of some species can be frozen [cyprinid fish: 5, toads: 19, poecillid fish: 60, frogs: 61], while freezing results in the deactivation of alarm cues in others [crayfish: 44, gobiid fish: 62].

Since the chemical structure of alarm cues is unknown, quantifying the concentration of these cues is imprecise. Researchers are left to guess alarm cue concentrations and attempt to standardize them by documenting the alarm cue collection methodology (e.g., whole-body maceration, cut sites, homogenized epidermal segments) and the ratio of individuals to water in which the cues are diluted [23,29,63,64]. Even forming cues based on the surface area or weight of the individual is not necessarily precise because intrinsic and extrinsic properties (e.g., diet quality, breeding condition, group familiarity) of those releasing the cues potentially alter the alarm cue potency in an individual [1,63,65–69]. For example, alarm cues from fish on a higher quality diet were found to trigger a greater antipredator response in conspecifics even after correcting for size (i.e., collecting cues from a standardized surface area), likely due to increased alarm cue production and thus concentration per area of skin [65,66]. This makes it difficult to establish the gradation in antipredator responses towards alarm cues and the cue concentrations are often highly variable between studies. Furthermore, since these cue concentrations cannot be measured precisely at a chemical level, the only method to determine the lower threshold and upper plateau of alarm cue responses and to help approximate ecological relevance, is through “trial-and-error” with follow-up studies that use modified methodology or dilutions [17,37]. For example, early research involving fathead minnow (*Pimephales promelas*) alarm cues used a concentration of 1 cm^2^ of homogenized skin tissue diluted in 6.9 mL water [70]. Then, in later studies, the concentration was modified to a ratio of 1 cm^2^ of homogenized skin tissue in 120 L of water [17], with the two different concentrations both eliciting overt antipredator responses in exposed conspecifics. Indeed, after enough research trials alarm cue preparation can become standardized, however, the aforementioned differences in alarm cue production locations and chemical properties makes it unlikely to be extrapolatable across classes or even across species [1]. Further complicating alarm cue quantification is the variation in exposure concentration [44,71–73] (for a summary, see Table 1). Once alarm cue stock solutions are formed, individuals are exposed to the cues when they are administered into the tank water [44,72–74]. If individuals are close enough to the cue administration source, they could be exposed to the alarm cue concentration of the stock solution. Alternately, if the cue administration source is further away, the cues could become further diluted in the tank water before the test subject detected them. Given the importance of alarm cue concentration in driving immediate antipredator responses and alarm cue learning [17,19,23,75], this is an important factor to consider when interpreting results.

**Table 1:** Crayfish alarm cue concentrations in various research studies.

Stock solution concentration	Exposure concentration	Corrected concentration	Species	Reference
1 crayfish/ 400 mL water	20 mL cue/ 12.5 L water (1.6 mL cue/ L)	0.0040 crayfish/ L water	*Faxonius virilis*	[79]
1 crayfish/ 150 mL water	10 mL cue/ 8 L water (1.25 mL cue/ 1 L water)	0.0083 crayfish/ L water	*Faxonius propinquus*	[72]
1 crayfish/ 150 mL water	Unclear	—	*Faxonius rusticus, F. virilis, Procambarus clarkii, Austropatmobius pallipes*	[14]
1 crayfish/ 150 mL water	5 mL cue/ 1.5 L water (3.33 mL cue/ L)	0.0222 crayfish/ L water	*P. clarkii, Faxonius limosus, A. pallipes*	[71]
1 crayfish/ 150 mL water	10 mL cue/ 15 L water (0.67 mL cue/ L water)	0.0044 crayfish/ L water	*F. rusticus, F. virilis*	[29]
1 crayfish/ 200 mL water	10 mL cue/ 15 L water (0.67 mL cue/ L water)	0.0033 crayfish/ L water	*P. clarkii*	[44]
Unclear	N/A – Flow through system	—	*F. virilis*	[80]
1 crayfish/ 100 mL water	Unclear	—	*F. rusticus*	[74]
1 crayfish/ 1 L water	500 mL cue/ 7 L water (71.42 mL cue/ L)	0.0714 crayfish/ L water	*F. virilis*	Exp 1 [78]
~0.15–3 crayfish/ 1 L water (dilution experiment)	500 mL cue/ 7 L water (71.42 mL cue/ L)	0.0107-0.2142 crayfish/ L	*F. virilis*	Exp 2 [78]
1 crayfish/ 30 mL water	1 mL cue/ 5.7 L water (0.18 mL cue/ L)	0.0058 crayfish/ L water	*F. virilis*	[81]
1 crayfish/ 400 mL water	N/A – Multiple administration points within a tank to create a gradient	—	*F. rusticus*	[4]
1 crayfish/ 400 mL water	20 mL cue/ 8.82 L water (2.27 mL cue/ L)	0.0057 crayfish/ L water	*F. rusticus*	[73]
1 crayfish*/ 10 L *5 washed cut sites	20 mL cue/ 10 L water (2.00 mL cue/ L)	0.0002 crayfish/ L water	*F. virilis*	Lowest concentration that elicited a response in this study

First, crayfish are crushed to release alarm cues, and the collected solution is diluted in water to yield the alarm cue “stock solution concentration”. A certain volume of this alarm cue stock solution is then administered into the test tank water which contains the test subject. This dilutes the alarm cues in the given volume of water in the test tank, yielding the “exposure concentration”. To account for the variability in both the stock and exposure concentrations and allow for approximate comparisons across studies, we used these values to calculate the “corrected concentration” which is the quantity of crushed crayfish per litre of water to which the test subjects were exposed (stock solution concentration × exposure concentration). However, this is a rough calculation as it does not take into account the size of the individual used to form the cues (because reported as weight in [4,44,73,78,79], as carapace length in [14,29,72,80,81] and unreported in [71,74]) nor other intrinsic or extrinsic factors that could alter cue potency between individuals (e.g., diet quality, breeding condition) [65,69]. All alarm cues were formed by crushing a whole crayfish unless otherwise stated.

Even though crayfish alarm cues have been studied since their characterization in this species in 1994 [76], there is a lack of consistency in cue concentrations and there is limited evidence even supporting a graded response to these cues [77,78]. At present, it is a standard practice to form alarm cues by completely crushing one whole crayfish, straining off the fluids, and diluting it to yield a final concentration of 1 crushed individual in 100–400 mL of water [4,44,72–74]. However, these cues are then administered into test tank water to yield exposure concentrations that range between 0.67–3.33 mL alarm cues per 1 L of test tank water [44,71–73]. This wide range in concentrations for both the alarm cue stock solution and the exposure concentration can compound, resulting in high variability in the final cue concentration to which the test subject is exposed. To allow for a better comparison of values, one method to account for both the variability in the stock solution and exposure concentrations is to mathematically correct the values by multiplying the alarm cue stock concentration (i.e., number of crayfish crushed/ volume of water into which they are diluted in litres) with the exposure concentration (i.e., volume of stock alarm cue solution administered in litres/ volume of the tank into which the cue is administered in litres). This yields a value that represents the quantity of crushed crayfish per litre of water to which a conspecific is exposed that we refer to as the “corrected concentration”. This measurement is a coarse estimate of concentration as the cue concentrations likely vary with other factors such as species and diet, as previously discussed [47–49,65,66], and does not account for the size of the crayfish crushed to form the cues. Indeed, while studies report the “number” of crayfish used to form alarm cues, there is variability in the size of the crayfish which is challenging to correct for as it is reported as either weight [4,44,73,78,79] or carapace length [14,29,72,80,81], or sometimes is undocumented [71,74]. Nonetheless, despite its imprecision, our correction calculation does makes the variability in alarm cue concentrations across research studies apparent as crayfish are exposed to anywhere between 0.004 and 0.022 of crayfish cue per 1 L of water [44, 71, 72, 73; for a summary, see Table 1].

Not only is there high variability in alarm cue concentrations in laboratory studies, but it is possible that the concentrations used are in the upper range (much like the early research conducted in fathead minnows) where antipredator responses could plateau, and could be more potent than is ecologically relevant. This would make subtle trade-offs between antipredator responses go undetected and could lead to incorrect conclusions about responses in natural populations. The current standard crayfish alarm cue methodologies use cues from whole crushed crayfish while laceration or bite wounds also likely occur in nature and would release much lower quantities of alarm cues to which it would be adaptive for prey to respond [82]. The purpose of this current study was to evaluate the threat sensitive predator avoidance hypothesis in crayfish by assessing their antipredator response towards alarm cues of different concentrations. Additionally, we wanted to gain a better understanding of the lower limit of their response range as it could help direct ecologically relevant research by providing insights into predator-prey interactions in nature and indicating the levels at which trade-offs are likely most apparent. To do this, we tested a different methodology to prepare and dilute the cues than is currently being used which could not only help standardize concentrations across studies but could also reduce the number of animals sacrificed for this research area. In experiment 1, northern crayfish (*Faxonius virilis*) alarm cues were prepared using methodology which had been modified from the current standard whole-body crushing procedure. Instead, five cut sites were flushed with water to collect the hemolymph-derived cue to mimic an injury scenario where a lower level of alarm cues would be released [82]. Crayfish were then tested for their antipredator response to conspecific alarm cues of three different concentrations (cues in 100–200 mL water) or a water control. The exposure concentration was standardized as 20 mL of cue was administered into 10 L of tank water containing the test subject. The crayfish were also tested for their response to frozen-thawed alarm cues – a process which does not interfere with alarm cue responses in fishes and amphibians [60,61], but potentially deactivates them in crayfish [44]. To expand on the findings of this initial study, experiment 2 tested the antipredator response of crayfish to frozen-thawed conspecific alarm cues of three additional concentrations, up to a dilution of one crayfish (i.e., five washed cut sites) in 100 L, compared to a water control. We predicted that the crayfish antipredator response would decrease with a decreasing alarm cue concentration and that they would not respond to frozen-thawed alarm cues, to be consistent with previous findings [37,44,83].

## Methods

### Test species

Northern crayfish (*Faxonius virilis*) were the chosen test subject as they, along with other crayfish species, are a common crustacean in laboratory studies [4,44] and have ecological importance as an ecosystem engineer in shallow slow-moving aquatic environments [84]. During the summer of 2022 (experiment 1) and 2023 (experiment 2) wild crayfish were collected and transported under the Saskatchewan Ministry of Environment Academic Research Permits 21AR027f and 23AR009fp. Individuals were captured from the South Saskatchewan River near the Clarkboro ferry crossing (13 km northeast of Saskatoon; latitude = 52.32054, longitude = −106.45760) by moving rocks and using dip-nets. The crayfish were maintained in lab in individual 5-L clear plastic tanks, each of which was filled full of dechlorinated tap water and contained gravel at a depth of 1.5 cm, an air bubbler, and a shelter (4” diameter PVC pipe cut in half). They were fed alfalfa pellets twice weekly and frozen-thawed tilapia once weekly. Three times a week, a 50% water change was also performed. Prior to testing, the crayfish were housed in the lab for a minimum of one week, and had been fed tilapia a minimum of two times, allowing them to recognize the food odour cues [85] which are an important component of the antipredator behavioural assay used in the following experiments.

### Ethical note

All the work performed in these studies, including animal husbandry, euthanasia, and testing, was equivalent to the guidelines set by the Canadian Council on Animal Care (CCAC) for fishes [86], as the CCAC does not have guidelines established for aquatic invertebrates.

#### Euthanasia.

Crayfish euthanasia was performed using a two-step method to minimize pain and distress as per CCAC and the American Veterinary Medical Association (AVMA) guidelines as well as the University of Saskatchewan’s University Animal Care Committee Euthanasia of Aquatic Animals Standard Operating Procedure #E201 [86–88]. First, they underwent a 10 min cold-shock anesthesia following the methodology of Driscoll et al. [81]. Second, they were euthanized using the splitting procedure (i.e., midline incision) [88] which is an acceptable alternative to chemical euthanasia [87] as the latter can interfere with behavioural alarm cue studies [89]. All euthanasia was performed with a minimum of two people present thereby ensuring proficiency, consistency, and that assistance was provided when needed.

### Experimental design

#### Experiment 1 – Alarm cue concentration and preparation methodology.

During a single testing phase, crayfish were exposed to one of five test cues which included a water control or conspecific alarm cues that were a high concentration, medium concentration, low concentration, or frozen (high concentration).

#### Experiment 2 – Alarm cue dilutions.

Experiment 2 also consisted of a single testing phase where crayfish were exposed to 1 of 5 test cues. Based on the results of the first experiment, the same high concentration alarm cue was used, as well as a 10x dilution, a 100x dilution, and a 1000x dilution and a water control. All alarm cues were frozen-thawed rather than fresh thereby reducing animal use as cues did not have to be made fresh daily.

### Alarm cue preparation

#### Alarm cue stock solution.

Randomly selected crayfish were used for alarm cue production (carapace length: mean ± SD = 31 ± 3 mm, approximately 62 mm total length in experiment 1; 34 ± 3 mm, approximately 70 mm total length in experiment 2). To mimic an unsuccessful predation event (e.g., bite or laceration wound), cues were collected from crayfish that were cross sectioned at five points rather than through the current standard method of completely crushing the individual [4,44,78]. The cut sites were located on the palm of both the right and left chelae as well as between pleonal somites 1–2, 3–4, and 5–6. To collect the alarm cues, both sides of each incision site was flushed with 10 mL of water and filtered to remove particles, yielding a base stock solution of 1 crayfish per 100 mL water. Antipredator responses in Northern crayfish have been documented at alarm cue stock solution concentrations ranging from 1 crushed crayfish per 100–400 mL water [4,44,80]. Since our study involved collecting cues from only cut sites, we selected the upper end of this range in concentrations (i.e., dilution into 100 mL water) to use as our base alarm cue concentration.

#### Alarm cue dilutions.

For the high concentration alarm cues, the stock solution (i.e., 1 crayfish/ 100 mL water) was used for both experiments to keep our results comparable. In experiment 1, the medium and low concentrations were formed by taking the stock alarm cue solution and adding water to reach a final concentration of 1 crayfish per 150 mL and per 200 mL respectively. For experiment 2, a series of 10x, 100x, and 1000x dilutions were created by adding water to the high concentration AC stock solution to yield final concentrations of 1 crayfish per 1 L, 10 L, and 100 L respectively. Frozen alarm cues were prepared by freezing the cues immediately after formation at −20°C for 24 h (experiment 1) or for approximately 1 month (experiment 2). They were then thawed before being diluted (if applicable) and used for testing. All cues were used within 5 h of collection or post-thaw in experiment 1 and within 3.5 h of thawing in experiment 2. A minimum of 3 crayfish alarm cues were pooled per batch of cue, with both sexes represented in each batch, thereby producing a minimum of 300 mL of stock solution per batch.

### Testing procedures

Randomly assigned crayfish received one of the five testing cues and were monitored for their antipredator response using a well-established behavioural assay [79,90]. It consisted of a 5 min pre-test cue period (S1a Video) and a 5 min post-test cue period (S1b Video) where the crayfish’s behaviour was video recorded (camera: Akaso V50X; resolution 1080p, 30fps).

First, individuals were moved into a 35-L glass tank (50 x 26 x 26.5 cm) containing 10 L of water, a 1-cm layer of white gravel, and a 10-cm long white U-shaped shelter (4” diameter PVC pipe cut in half) (S1 Fig). The testing tank was surrounded by black plastic to keep the crayfish visually isolated. After a 30 min acclimation period, the pre-test cue period initiated with syringe-administration of 5 mL food odour followed by 20 mL water into the tank thereby stimulating foraging activity in the normally inactive crayfish (S1a Video). The food odour consisted of soaking 1 g of macerated tilapia in 100 mL of dechlorinated tap water before filtering it for use, as described by Acquistapace et al. [44]. The tilapia was placed in the water and macerated with the blunt end of a syringe plunger for 15 sec then stirred 2–3 times during the 1 h soaking period. Since 5 mL of the food odour solution was administered into 10 L of water, it yielded an exposure concentration of 1 g soaked food/ 1 L water, similar to those used in prior research [44]. The water administration that followed the food odour served as a procedural control to ensure the same amount of disturbance (i.e., same volume) occurred at the onset of both the pre-test cue and post-test cue period.

After completion of the 5 min pre-test cue period, an additional 5 mL of food odour and 20 mL of the test cue (i.e., alarm cues or water) were sequentially syringe-administered into the tank and the 5 min post-test cue period was initiated. While there is high variability in the exposure concentration of test subjects to alarm cues (as previously explained), this ratio was similar to those used in previous studies [73,79]. All cues were administered just above the water with a drip hose attached to a long rod to minimize visual disturbances. The location of the freely moving test subjects could bias the concentration of cues they detect, with those close to a fixed drip hose receiving a concentrated solution while those further away receiving a very dilute solution. Thus, the cues were administered along a lengthwise and widthwise transect that passed within 5 cm of crayfish.

Freezing is a well documented antipredator behaviour in crayfish, therefore, a decrease in activity can serve as an indicator for this response (S1b Video) [90,91]. However, inactivity can also signal decreased food-motivation or moulting [92]. Therefore, the re-administration of food odour at the start of the post-test cue period ensured that any decreases in activity were not simply a result of dissipated food stimuli. Likewise, crayfish were not tested if they moulted within 4 days prior to testing and were excluded from the dataset if they moulted within 24 h post-testing. Furthermore, to ensure crayfish were active prior to the post-test cue administration (to allow for decreases in activity to be observed), they were visually monitored and we only proceeded with the post-test cue exposure if crayfish were active for a minimum of 30 sec during the pre-test cue period. Those who failed to meet this minimum activity level were re-tested a minimum of 24 h later, up to a maximum of four times.

Testing occurred indoors, between 10:00 and 22:00 h, at water temperatures of 18 ± 3°C under fluorescent lighting. To avoid biases, the order of the treatments tested was randomized. The sex and size (carapace length: measured at dorsal midline from the tip of the rostrum to the base of the carapace) of all crayfish was recorded immediately after testing and accounted for in the analysis as described below. We did not record crayfish reproductive status and likely tested both those in reproductive (form I) and non-reproductive (form II) states. Nonetheless, since testing was randomized, there should be an approximately equal proportion of crayfish in the two reproductive forms in each treatment group. Thus, this experiment makes conclusions on general crayfish behaviour across reproductive states. A total of N = 130 and N = 126 crayfish were successfully tested, yielding n = 24−28 and 20−20 per treatment in experiment 1 and experiment 2 respectively.

### Video analysis

The activity level of each crayfish was quantified as “time spent moving” (in sec) from video-recorded trials using Noldus Ethovision XT video analysis software (version 16, Noldus Information Technology, Wageningen, Netherlands). Crayfish were defined as moving when they crossed a start velocity threshold of 0.40 cm/s and defined as not moving when they crossed a stop velocity threshold of 0.30 cm/s, at a sample rate of 3 Hz. Preliminary trials established that these metrics captured all crayfish walking parameters. Crayfish within the shelter (defined as a specific zone in the video analysis software during data extraction) were defined as not moving. Track smoothing was used to minimize detection errors such as “jumps” from the test subject (i.e., the crayfish) to other objects in the test arena (e.g., water surface ripples, light reflections), by correcting the tracking pathway of the test subject to the last position point if they moved more than 5 cm during one 0.33 sec sample. Track smoothing also minimizes over-estimates that can be made to the movement parameter if body shifting of stationary crayfish is detected as locomotory movement. Specifically, the tracking pathway of the subject is corrected to the last position point if they moved less than 0.15 cm during one sample (i.e., 0.33 sec).

Shelter use is also a common antipredator behaviour of crayfish [4,7,81], thus, we intended on analyzing that behavioural outcome. However, the crayfish in our test population did not appear to use the half-pipe style shelter in our behavioural assay, as has occurred in other study populations [3]. In experiment 1, only n = 6 and n = 3 crayfish occupied the shelter for longer than 10 sec (i.e., not just walking through) in the pre- and post-test cue periods respectively. Similarly, in experiment 2, only n = 11 (pre-test cue period) and n = 11 (post-test cue period) crayfish used the shelter. Thus, shelter use behaviour was expressed by <9% of our test subjects and was dropped from our analysis.

Detection settings were individually established for each trial using “dynamic subtraction”. This feature uses the difference in contrast between the subject and the background to track the test subject, and updates this information throughout the trial to allow for changes to the background environment that could otherwise interfere with tracking, such as water ripples. We required a minimum successful detection rate of 90% for the data to be used which was calculated using the time the computer successfully tracked the crayfish. If the detection rate was below this threshold, the detection settings (e.g., contrast) were adjusted and the video was reanalyzed up to a maximum of 3 times. Across both experiments, 7–15 trials failed to reach this detection level after initial analysis, and 1–3 videos were unable to reach this detection level after reanalysis. Additionally, we required that crayfish moved a minimum of 30 sec during the pre-test cue baseline to ensure changes in behaviour could be observed if present. To avoid observation biases, researchers were blind to the treatments when establishing detection settings and performing any reanalysis.

### Statistical analysis

#### Experiment 1 – Alarm cue concentration and preparation methodology.

To ensure that crayfish had a similar baseline activity level, the pre-test cue time spent moving was analyzed using an ANCOVA, thus testing for any behavioural biases between the different cue type groups (i.e., fixed factor) while accounting for crayfish sex (i.e., random factor) and size (i.e., covariate) (model: y = test cue + sex + carapace length). To evaluate the change in activity, we calculated the proportional change in time spent moving from the pre-test cue baseline ([pre – post]/ pre). A second ANCOVA (same model) was performed on this data. There was no significant effect of crayfish sex or carapace length on the pre-test cue baseline activity level (sex: F_1,123_ = 0.41, p = 0.52; carapace length: F_1,123_ = 3.25, p = 0.07) nor on the proportional change in activity (sex: F_1,123_ = 0.84, p = 0.36; carapace length: F_1,123_ = 0.63, p = 0.43). This indicates that crayfish sex and size did not affect crayfish activity, therefore, they were removed from all subsequent analyses for clarity. Both the pre-test cue activity and the proportional change in activity were re-analyzed with a 1-way ANOVA followed by post-hoc Tukey tests to determine whether there were differences in activity between the 5 different test cues.

Residual plots revealed minor deviations in normality for both the pre-test cue baseline data (1 out of 5 samples) and the proportional change in activity data (2 out of 5 samples). Since ANOVAs are robust against deviations from normality [93], we proceeded with the analyses. The assumption of equal variances (Levene’s test: pre-test cue activity, F_4,125_ = 0.26, p = 0.90; proportional change in activity, F_4,125_ = 1.77, p = 0.14) and homogenous slopes (ANCOVA interaction of test cue × carapace length: pre-test cue activity, F_4,119_ = 0.61, p = 0.66; proportional change in activity, F_4,119_ = 0.46, p = 0.76) between the 5 treatment groups were met. All analyses were performed using SPSS 25 (SPSS Inc, IBM).

#### Experiment 2 – Alarm cue dilutions.

The pre-test cue time spent moving was analyzed using a 1-way ANOVA, to determine if there were any differences in the baseline activity level between the groups assigned to the five different cue types. The proportional change in time spent moving was calculated as described in experiment 1. A 1-way Welch’s ANOVA was used when analysing the proportional change data for differences in responses to the five test cues, as not all the treatment groups had equal variances (Levene’s test: F_4,121_ = 5.99, p < 0.001), unlike the pre-test cue movement data where this assumption was met (Levene’s test: F_4,121_ = 1.51, p = 0.21). Following this analysis, a Games-Howell post-hoc test was used to further define the differences in the proportional time spent moving between the five different test cues. Inspection of the residual plots revealed that two samples have a mild deviation from normality but, as in the first experiment, a parametric analysis was still used. All analyses were performed using SPSS 25 (SPSS Inc, IBM).

One severe outlier in the alarm cue 1000x dilution treatment group was identified and removed before any statistical analyses were performed. Severe outliers were defined as datapoints falling outside an interval of [Q1 – (3)(IQR)] and [Q3 + (3)(IQR)] where Q1 is the 25th percentile, Q3 is the 75th percentile, and IQR is the interquartile range.

## Results

### Experiment 1: Alarm cue concentration and preparation methodology

**Fig 1:**
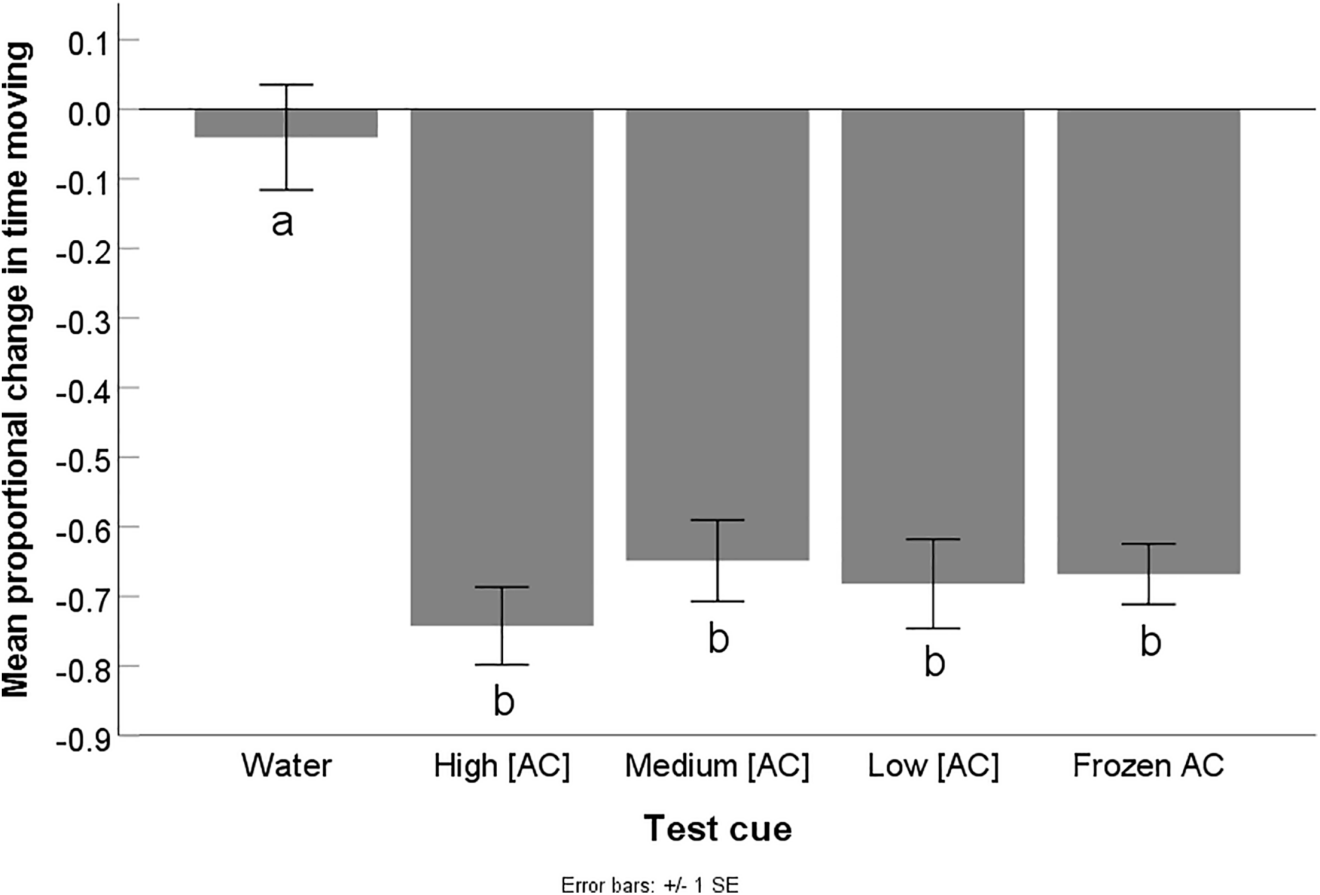
Results for experiment 1: Alarm cue concentration and preparation methodology. Mean (± SE) proportional change in time spent moving from the pre-test cue baseline (pre – post/ pre) of crayfish exposed to one of five different test cues including water (water), high concentration alarm cues (high [AC]), medium concentration alarm cues (medium [AC]), low concentration alarm cues (low [AC]), and frozen high concentration alarm cues (frozen AC)(n = 24-28 per treatment). A decrease in activity indicates an antipredator response. Different letters indicate statistical significance at α = 0.05.

There were no differences in the pre-test cue baseline activity level of the crayfish assigned to receive the different cue type (F_4,125_ = 0.76, p = 0.56), indicating that no pre-existing behavioural biases were present between the treatment groups. The crayfish’s mean proportional time spent moving differed depending on the test cue used (F_4,125_ = 24.23, p < 0.001). Those exposed to any of the alarm cue treatments had a greater decrease in activity compared to crayfish exposed to water (water vs each alarm cue treatment: all p < 0.001) indicating they underwent an antipredator response to alarm cues, compared to the water control. Additionally, all alarm cue treatments elicited similar changes in activity when compared to each other (all 0.82 < p < 0.99), representing an equivalent antipredator response to these cues (Fig 1).

### Experiment 2: Alarm cue dilutions

**Fig 2:**
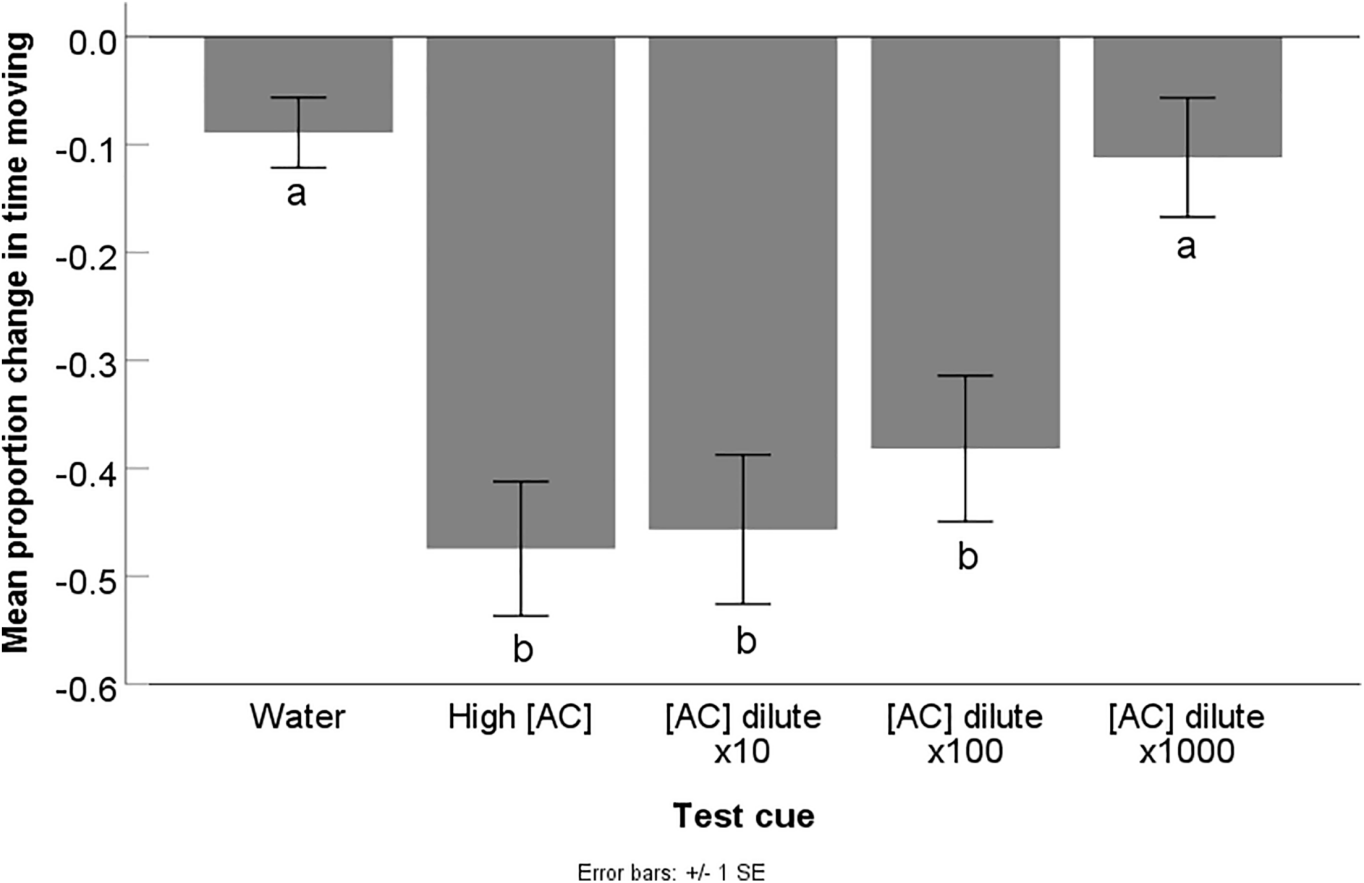
Results for Experiment 2: Alarm cue dilutions. Mean (± SE) proportional change in time spent moving from the pre-test cue baseline (pre – post/ pre) of crayfish exposed to one of five different test cues including water (water) and high concentration alarm cues (high [AC]) as well as alarm cues that had been diluted by a factor of 10 ([AC] dilute x10), 100 ([AC] dilute x100), and 1,000 ([AC] dilute x1000)(n = 20-28 per treatment). A decrease in activity indicates an antipredator response. Different letters indicate statistical significance at α = 0.05.

The pre-test cue baseline activity level of the crayfish from different treatment groups did not differ (F_4,121_ = 0.44, p = 0.78), indicating that there were no behavioural biases between the treatment groups prior to cue administration. The mean proportional time spent moving of the crayfish differed depending on the test cue exposed (F_4,58.123_ = 12.84, p < 0.001) (Fig 2). Compared to the water control, crayfish had a significant decrease in activity when exposed to the high alarm cue concentration (p < 0.001) as well as dilutions of 10x (p < 0.001) and 100x (p = 0.004), indicative of an antipredator response. The response to these three alarm cue concentrations did not differ from one another (0.85 < p < 0.99). On the other hand, the behaviour of crayfish exposed to the alarm cues that had a dilution of 1000x did not differ from that of crayfish exposed to water (p = 0.77). Furthermore, this 1000x alarm cue dilution elicited a lower change in activity compared all the other concentrations (high vs 1000x: p = 0.001; 10x vs 1000x: p = 0.004, 100x vs 1000x: p = 0.045) (Fig 2).

## Discussion

The findings of these two experiments show that crayfish are capable of responding to previously frozen alarm cues (experiment 1) and that they respond to concentrations that are highly diluted compared to the standard alarm cue concentrations used in current crayfish research (experiment 2) (Table 1). However, neither experiment found evidence to support a graded antipredator response to alarm cues. All these findings have implications for research that will (1) help guide methodology in studies examining antipredator responses involving aquatic chemical cues, (2) contribute to our understanding of predator-prey interactions that are ecologically-relevant, and (3) improve animal welfare.

Crayfish antipredator behaviour towards frozen-thawed alarm cues is a response that is undocumented to date. Moreover, two studies [44,79] have found evidence that crayfish fail to respond to previously frozen alarm cues. In the paper by Hazlett [79], he comments that crayfish do not respond to previously frozen alarm cues through personal observations, so no speculations on methodology can be made. Conversely, the experiment performed by Acquistapace et al. [44] follows methodology similar to this experiment 1 whereby the alarm cues are frozen immediately after preparation at −20°C for 24h, however, two key differences exist. The first difference is that Acquistapace et al. [44] tested red swamp crayfish (*Procambarus clarkii*) while this current study tested northern crayfish, therefore, interspecific variability could be responsible for the differing responses towards frozen-thawed alarm cues. Potentially, northern crayfish hemolymph could possess some molecules that preserve the activity of alarm cues when frozen which red swamp crayfish do not possess. Indeed, northern crayfish occupy a more northern range compared to red swamp crayfish [94,95]. The second difference is that the prior study froze extracted hemolymph while the present study froze a wash of cut-sites, thus more chemical compounds would be included in the latter solution. Acquistapace et al. [44] did validate that alarm cues are present in hemolymph and can trigger an antipredator response in conspecifics, so the problem is not that the hemolymph alone does not trigger an antipredator response. Rather, there is the potential that other chemicals located elsewhere in the crayfish interact with compounds in the hemolymph which stabilize it during the freezing process, thus preserving the bioactivity of the alarm cue compounds. These two hypotheses are supported by a different study by Hazlett [85] whereby northern crayfish were frozen whole and later thawed and used to produce alarm cues which elicited an antipredator response in exposed conspecifics. A final aspect of the Acquistapace et al. [44] study is that the experiment using the frozen hemolymph cues had no positive control. While they validated that hemolymph elicited an antipredator response initially, in their subsequent experiment involving frozen cues no crayfish in any of the treatment groups underwent an antipredator response, irrespective of alarm cue processing. Therefore, there could have been an inherent issue with the batch of alarm cues or test crayfish used in that specific experiment that went undetected in that experimental design resulting in the lack of response.

While the ability to freeze alarm cues may appear to be a methodology nuance, it has significance for researchers studying aquatic chemical ecology and antipredator responses ranging from morphology to behaviour. Different intrinsic factors of individuals producing alarm cues are known to elicit antipredator responses of different magnitudes in exposed conspecifics [1]. For example, individuals that have a better body condition trigger greater antipredator responses (when controlling for size), likely because they have more nutrients so more alarm cues can be produced per area of tissue [65]. Similarly, the size of the individuals releasing the cues affects conspecific antipredator behaviour, either due to alterations within the alarm cue chemical structure, or through the presence of other chemical cues (e.g., odour) that give information on size [63,96]. Antipredator behaviour can also be altered given intrinsic properties of the individual detecting cues (e.g., size, age, starvation, sex) or extrinsic environmental factors (e.g., pollutants, group size, risk level), causing further variation in behavioural responses [27–29,97–99]. Given the variability that can be associated with alarm cue responses, researchers seek to minimize this variation to allow them to detect often subtle behavioural patterns [100,101]. One way in which this can be achieved is to minimize the variability caused by cue preparation across trials. If large batches of alarm cues with multiple crayfish can be formed and then preserved over time through freezing, the cue mixture can be used throughout the trial. This washes out the effects of individual variation from the cue donors and yields more consistent research results.

Experiment 2 found that crayfish still responded with an overt antipredator response when alarm cues had a concentration of 1 crayfish diluted in 10 L of water, yielding a final corrected exposure concentration equivalent to 0.0002 crayfish cue per 1 L water (calculation: alarm cue stock solution concentration × exposure concentration). This is 20-110x more diluted than the concentrations used in previous research where the corrected values range from 0.004–0.022 crushed crayfish cue per 1 L of water (Table 1) [44,71–73]. Furthermore, that is not accounting for the methodology differences when forming the cue. The current standard method is to collect cues from a whole crayfish [44,71–73], which would yield a higher concentration of cues than our procedure whereby we collected cues from five cut sites on a single crayfish. Thus, this procedural difference would further amplify the difference in alarm cue concentrations beyond 20-110x, although the exact magnitude is unquantifiable.

Establishing the range of alarm cue concentrations to which crayfish respond is important for research in aquatic chemical ecology as researchers consider the effects of the threat sensitive predator avoidance hypothesis [15]. In accordance with this hypothesis, various species [17–21] exhibit graded responses to alarm cues, often up to a certain concentration where responses then plateau as they reach maximal expression [10,31–33]. However, the evidence of an alarm cue concentration gradient effect in crayfish is less conclusive, both in this present study, and in previous experiments. A study in northern crayfish found some evidence of a graded antipredator response towards alarm cues, however, the experimental design lacked the fine-scale concentration variation to make this conclusive [77]. In a flume, the center area had a high concentration of alarm cues that gradually dissipated to the periphery and the crayfish were found to preferentially occupy the edges [77]. While the crayfish evidently avoided the high concentration alarm cue, they did not record whether the crayfish progressively increased their time spent in the periphery thereby supporting the graded response hypothesis. In another study, there was a trend of decreased northern crayfish activity (i.e., antipredator response) with an increase in alarm cue concentration, however, the effect was nonsignificant [78]. Conversely, one experiment suggested that northern clearwater crayfish (*Faxonius propinquus*) have ungraded antipredator behaviour towards alarm cues, instead performing an all-or-nothing response after a given concentration is reached [72].

One explanation for these variable results on crayfish alarm cue response gradients could be that the concentrations of cue they were using to form their gradients were too high (i.e., in the range where responses are maximized and plateau). If the alarm cues are so potent that the crayfish are exhibiting a maximal antipredator response at even the lowest alarm cue concentration, there would be no observed increase in response with an increase in concentration. Indeed, the study by Bouwma and Hazlett [72] crushed one crayfish in 300 mL to 15 L of water, yielding a corrected exposure concentration of 0.008 crayfish per 1 L water (after accounting for the cue volume administered into the testing tank). Ramberg-Pihl and Yurewicz [78] used 2–32 g of crushed crayfish diluted into 1 L of water. They reported that their study crayfish were approximately 10–15 g, therefore, these concentrations can be roughly estimated as 0.15–3 crushed crayfish in 1 L of water (i.e., the corrected concentration). Thus, the lowest concentration in both these studies are 40-50x higher than the lowest concentration that elicited an overt antipredator response in our study, especially since we obtained our cue from cut sites whereas these other studies diluted cues from a completely crushed crayfish. While our study also did not find a concentration gradient per se, the lowest alarm cue concentration we used (5 crayfish cuts washed into 100 L of water) elicited antipredator behaviour that was only marginally lower than responses elicited by alarm cues diluted into 10 L of water. Thus, one suggestion is that the concentrations used in our experiment were also high, surpassing a threshold at which the responses plateaued in a maximal antipredator response; this species of crayfish could still perform a graded response to alarm cues at concentrations between the cue stock solution dilutions in 10 L to 100 L of water. However, future research would be needed to confirm this hypothesis. Regardless, this knowledge is a significant contribution to the field of chemical aquatic ecology because alarm cues which are formulated in this range have a greater potential to detect subtle trade-offs between antipredator behaviour and other fitness-related activities which would be otherwise missed when potent risk cues are used.

Determining the lower limit of alarm cue concentrations to which crayfish respond provides insights on ecologically relevant quantities of these cues and how predator-prey interactions involving crayfish may occur in nature. As previously discussed, currently alarm cues are unquantifiable because the chemical structure is unknown and likely varies across species [44,46,52,55,56,58]. Consequently, there is a lack of knowledge on the quantity of alarm cues released during predator-prey interactions. In principal, successful predation events would involve whole-body injury that releases large amounts of alarm cues and unsuccessful predation events would involve bite or laceration wounds with less quantities of alarm cues [25,26]. However, many other factors influence the final concentrations of alarm cues that conspecifics typically detect in nature such as different predator hunting styles, population density (i.e., proximity to conspecifics), cue leakage rate from wound sites, water dynamics (e.g., flow rates), water chemical properties, degradation rates, and intrinsic or extrinsic properties of the individual releasing the cue (e.g., diet quality, breeding condition, group familiarity) [1,65,66,69,102–105]. Nonetheless, establishing the lower limit to which a given species does respond to alarm cues indicates a concentration that is ecologically relevant as it was adaptive for the population to overtly respond to the cue at that level. This study reveals that relevant cue concentrations in nature can extend 20-110x lower than those used in current research (see Table 1 for a summary). These dilute cue concentrations could occur as the chemicals dilute in the surrounding water, allowing conspecifics who are further away from the predation event to detect it and respond accordingly [1].

Dilute cue concentrations could also arise from minor injuries sustained during predator attacks [82]. Indeed, different predators have different hunting strategies and prey handling methods which could have different likelihoods of prey injury and result in different quantities of alarm cue release [105,106]. For example, fish predators such as smallmouth bass (*Micropterus dolomieu*), generally consume crayfish whole, but they have been observed to release crayfish after picking them up in their mouths, with the primary capture point being the carapace and abdomen [92]. However, injuries after this interaction are undocumented. Similarly, a study reported that crayfish escaped European perch (*Perca fluviatilis*) during handling in five out of six instances, with one individual escaping after being held in a perch’s mouth for 6 min [107]; again, wounds sustained by the crayfish were unreported. Nonetheless, injuries to the carapace, chelae, and abdomen that would result in hemolymph, and thus alarm cue, release have been observed in various field studies [82,108–111] as well as in the wild population of crayfish from which our test subjects were collected (personal observations). Indeed, a field study examining noble crayfish (*Astacus astacus*) found that 6% of males and 5% of females had visible damage to areas that would have resulted in hemolymph release (i.e., carapace, chelae) [82]. This is potentially an underestimate as injured individuals are at higher risk of mortality due to predation, cannibalism, or disease, thus, they could die before being documented [112].

Many of the injuries to crayfishes’ carapace, chelae, and abdomen are attributed to predation events [82], with large contributions likely from predators which can manipulate prey and consume it piecemeal, as is performed by terrestrial animals and birds [113,114]. One such crayfish predator is the raccoon, which has been observed in field studies to primarily consume the abdomen and thorax, leaving the remaining carcass on shore or submerged in the water where it would leak alarm cues [108,109]. Similarly, cranes and loons forage on prey such as crayfish by stabbing at them and have been known to tear them and remove the chelae before ingestion [115,116]. The attack methods and prey handling performed by these predators likely results in crayfish alarm cue release when predation is either successful or unsuccessful. A field experiment noted partial consumption of crayfish, however, they never specified whether it occurred with terrestrial predators (e.g., racoons, herons) or aquatic predators (e.g., fishes) [117]. Nonetheless, minor injuries and distant attacks are just initial hypotheses for the mechanism by which alarm cues become diluted in nature and require future predator-prey research to investigate.

The findings of our study have value for animal welfare as a goal of research is to minimize animal sacrifice while still achieving reliable results. As demonstrated by this study, crayfish alarm cues can be diluted in large quantities of water, so a single crayfish produces a large quantity of cues. Thus, by using this alternative concentration (1 crayfish to produce 10 L of alarm cues), fewer individuals are required to be sacrificed to yield the same volume of alarm cues, allowing researchers to support animal ethics initiatives. However, the ability to use frozen-thawed crayfish alarm cues, as identified in our study, is essential to gain the benefits of reduced animal sacrifice when using diluted cues. This is because current research suggests that alarm cues must be used within a few hours of formation or else they will degrade [44], and the sacrifice by that individual would be “wasted”. Since experiments generally last longer than this degradation window, multiple fresh batches of alarm cues must be made for a single experiment. However, if alarm cues can be frozen, then cues can be thawed as needed, allowing a single batch of alarm cues to be used over multiple days, thus reducing the number of individuals that need to be sacrificed.

Although it is basic, the information gained through our two experiments is important to aquatic chemical ecology research, a field which is increasing due to its importance for conservation efforts. As we alter natural environments at a rapid rate, we have a responsibility to the animals that live there to try and mitigate the impacts we have on these ecosystems. To do this, we must understand the threat-sensitive antipredator responses of animals to new predation threats (e.g., invasive species) or old threats in a changed environment. Typically, this is examined in lab and then the results are extrapolated into an ecological context and used to predict the effects of environmental change on populations. However, our attempts to mitigate environmental impacts can only be as effective as our research results are accurate. Therefore, methodology studies must be performed to validate experiments and to continually improve research techniques as well as lab-animal welfare, even if this validation is for concepts as simple as identifying cue concentration that are ecologically relevant.

## Supporting information

S1 Video Representative examples of crayfish behavioral responses. Excerpts from videos recorded during the experiment, specifically during (a) the pre-test cue period and (b) the post-test cue period of the antipredator behavioural test. The videos are of crayfish performing behavioural responses that are a representative example of (a) foraging (i.e., crayfish with a high activity level) and (b) a freezing antipredator response (i.e., crayfish with a low activity level).
(ZIP)

S1 Fig Testing tank diagram. A diagram of the tank used for the crayfish behavioural assay viewed from the (a) top and (b) side. The tank contained one U-shaped shelter made from a 4” diameter PVC pipe cut in half and cut to a length of 10 cm. It also contained white gravel at a depth of 1 cm and was filled with 10 L of water.
(TIF)

S1 File Raw data from experiments 1 and 2. An excel spreadsheet containing the raw data from the alarm cue concentration and preparation methodology experiment (exp 1; sheet 1) and from the alarm cue dilutions experiment (exp 2; sheet 2).
(XLSX)
