# Supplementary figures and images for: The power of concentration: Antipredator responses to diluted frozen crayfish alarm cues provide insights on ecologically relevant concentrations and updates to methodology
Source: PLoS One. 2025 Dec 31;20(12):e0340001. doi: 10.1371/journal.pone.0340001 (PMC12755739; doi:10.1371/journal.pone.0340001)

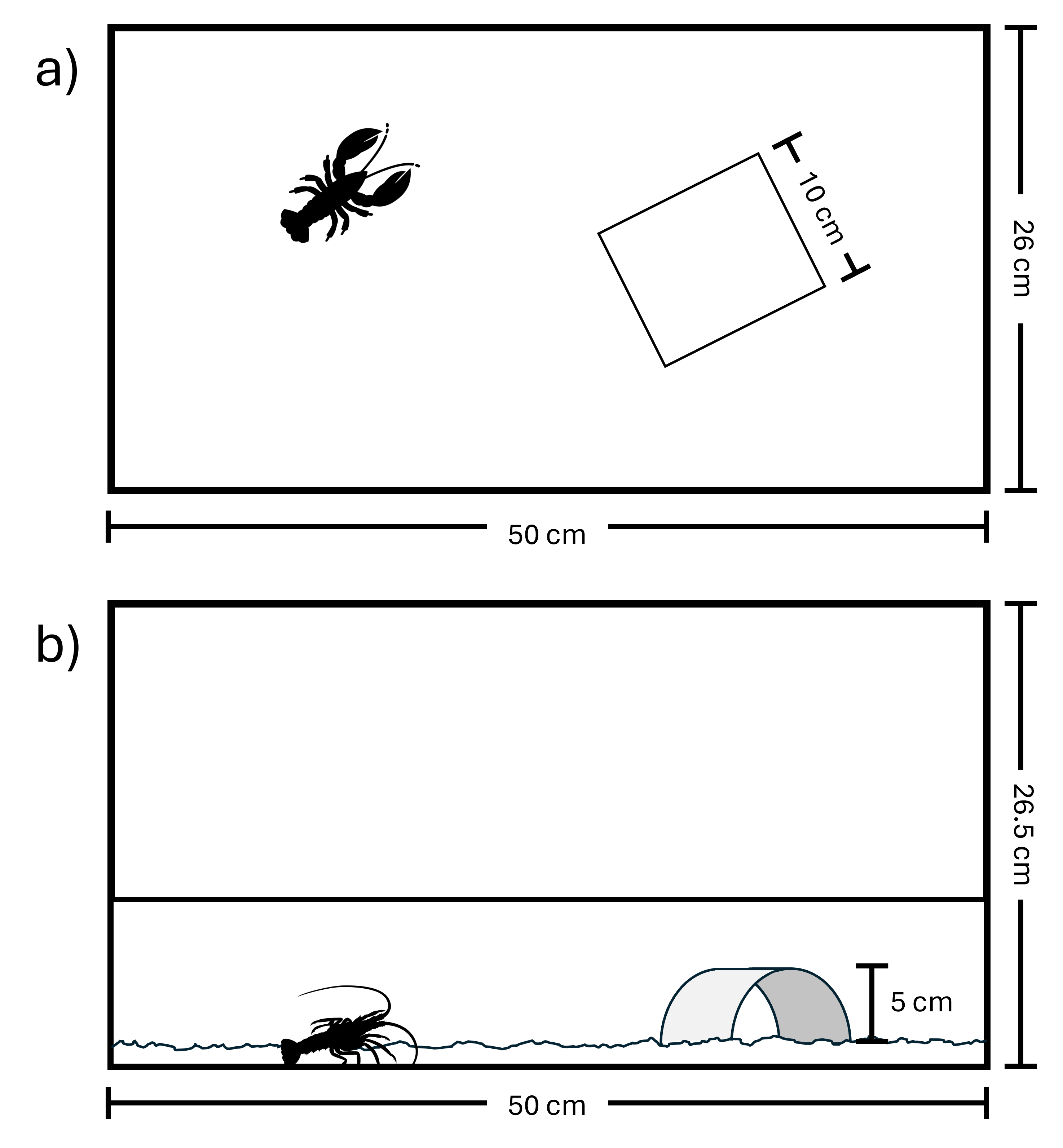

Supplement: S1 Fig — A diagram of the tank used for the crayfish behavioural assay viewed from the (a) top and (b) side. The tank contained one U-shaped shelter made from a 4” diameter PVC pipe cut in half and cut to a length of 10 cm. It also contained white gravel at a depth of 1 cm and was filled with 10 L of water. (TIF) [file pone.0340001.s002.tif]
